# Power to identify a genetic predictor of antihypertensive drug response using different methods to measure blood pressure response

**DOI:** 10.1186/1479-5876-10-47

**Published:** 2012-03-13

**Authors:** Stephen T Turner, Gary L Schwartz, Arlene B Chapman, Amber L Beitelshees, John G Gums, Rhonda M Cooper-DeHoff, Eric Boerwinkle, Julie A Johnson, Kent R Bailey

**Affiliations:** 1 Division of Nephrology and Hypertension, Department of Medicine, Mayo Clinic, Rochester, MN 55905, USA; 2 Division of Biostatistics, Department of Health Sciences Research, Mayo Clinic, 200 First Street S.W, Rochester, MN, USA; 3 Renal Division, Department of Medicine, Emory University School of Medicine, Atlanta, Georgia, USA; 4 Department of Medicine, University of Maryland School of Medicine, Baltimore, MD, USA; 5 Department of Pharmacotherapy and Translational Research and Center for Pharmacogenomics, University of Florida, Gainesville, FL, USA; 6 Department of Community Health and Family Medicine, University of Florida, Gainesville, FL, USA; 7 Department of Medicine, University of Florida, Gainesville, FL, USA; 8 Human Genetics and Institute of Molecular Medicine, University of Texas Health Science Center, Houston, TX, USA

**Keywords:** hypertension, blood pressure monitoring, antihypertensive drug therapy, beta-blocker, thiazide diuretic, plasma renin activity

## Abstract

**Background:**

To determine whether office, home, ambulatory daytime and nighttime blood pressure (BP) responses to antihypertensive drug therapy measure the same signal and which method provides greatest power to identify genetic predictors of BP response.

**Methods:**

We analyzed office, home, ambulatory daytime and nighttime BP responses in hypertensive adults randomized to atenolol (N = 242) or hydrochlorothiazide (N = 257) in the Pharmacogenomic Evaluation of Antihypertensive Responses Study. Since different measured BP responses may have different predictors, we tested the "same signal" model by using linear regression methods to determine whether known predictors of BP response depend on the method of BP measurement. We estimated signal-to-noise ratios and compared power to identify a genetic polymorphism predicting BP response measured by each method separately and by weighted averages of multiple methods.

**Results:**

After adjustment for pretreatment BP level, known predictors of BP response including plasma renin activity, race, and sex were independent of the method of BP measurement. Signal-to-noise ratios were more than 2-fold greater for home and ambulatory daytime BP responses than for office and ambulatory nighttime BP responses and up to 11-fold greater for weighted averages of all four methods. Power to identify a genetic polymorphism predicting BP response was directly related to the signal-to-noise ratio and, therefore, greatest with the weighted averages.

**Conclusion:**

Since different methods of measuring BP response to antihypertensive drug therapy measure the same signal, weighted averages of the BP responses measured by multiple methods minimize measurement error and optimize power to identify genetic predictors of BP response.

## Background

Although office blood pressure (BP) measurements remain the standard-of-care, averages of out-of-office measurements are more reproducible [1]. Out-of-office averages have also been reported to be more strongly correlated with subclinical target organ damage [2,3] and to better predict future cardiovascular disease events [4-6] than office measurements. Not surprisingly, BP responses to antihypertensive drug therapy are more precisely and accurately determined by out-of-office than office measurements, which are influenced by white coat and placebo effects [7,8]. Consequently, greater use of out-of-office methods of BP measurement has been advocated for clinical decision-making and research [1].

More individualized approaches to antihypertensive drug therapy may become possible if genetic polymorphisms are discovered that improve the ability to predict inter-individual differences in BP response [9]. Known predictors are limited to race, age, and plasma renin activity [10,11], which explain less than 50% of interindividual variation in BP response to single-drug therapy [12,13]. Most previous studies have attempted to identify genetic or non-genetic predictors of office BP response, which is not very reproducible and correlates only modestly with home and ambulatory BP responses [7,8,14-16]. Whether out-of-office measurements of BP response can improve the ability to identify predictors of BP response has not been demonstrated. Method-specific measurement errors could account for differences in the magnitude of and correlation between office, home, ambulatory daytime and nighttime BP responses [8]. However, an additional possibility is that different BP response signals are measured by the different methods.

Since different BP response signals may have different predictors, our first objective in the present study was to test the "same signal" model by determining whether known predictors of BP response, i.e., race, age, and plasma renin activity, depend on the method of BP measurement. We analyzed data from the Pharmacogenomic Evaluation of Antihypertensive Responses (PEAR) study, in which BP responses to single-drug therapy with atenolol or hydrochlorothiazide were measured by all four methods [7,8]. In this context, our second objective was to estimate signal-to-noise ratios and compare the power to identify a genetic polymorphism predicting BP response when measured by each method separately and by weighted averages of multiple methods.

## Methods

### Participants

The PEAR study [17]http://clinicaltrials.gov/ct2/show/NCT00246519 was approved by the Institutional Review Board at each site, and all participants gave informed consent. At an initial consent and screening visit, trained study personnel administered standardized questionnaires, performed a limited physical examination, and obtained blood and urine samples for testing to establish eligibility for participation [11]. Participants were provided an automated sphygmomanometer (MicroLife 3 AC1-PC, Minneapolis MN), the adequacy of which has been previously validated [18], and withdrawn from previous antihypertensive drug therapy. The device was set to measure BP in triplicate with each activation and to store the average systolic and diastolic BPs and the time of each set of measurements. Participants were instructed to take readings daily in the seated position, one set of three readings in the morning upon arising from bed and a second set in the evening just before retiring. At subsequent study visits (prior to randomization and at the end of therapy), an additional set of three readings was obtained seated (> 5 minutes) in the office using the home monitor. In addition, 24-hour ambulatory BP recordings were obtained at these visits using Spacelabs (Redmond WA) ambulatory monitors, model 90207, the adequacy of which has been previously validated [19]. Participants were instructed to conduct their usual daily activities while wearing the monitor, which was set to record BP four times per hour during the day (6 AM to 10 PM) and twice per hour during the night (10 PM to 6 AM). The average (± standard deviation) number of ambulatory measurements was 67 ± 10 during daytime hours and 15 ± 3 during nighttime hours.

At the end of the drug-free washout period, fasting blood samples were drawn in the seated position after ambulation for measurement of plasma renin activity [11]. To qualify for randomization, the average home diastolic BP in the previous week had to be ≥85 mmHg (consisting of at least five morning and five evening sets of readings) *and *the average office diastolic BP ≥ 90 mmHg. Participants received either atenolol or hydrochlorothiazide, starting at 50 mg or 12.5 mg daily, respectively, for two weeks, after which, if BP remained > 120/70 mmHg, the doses were increased to 100 mg or 25 mg daily, respectively, for six additional weeks.

### Statistical analysis

Analyses were performed with Statistical Analysis System software, version 9.1 (SAS, Raleigh-Durham NC). Statistical significance was defined a priori by *P *< 0.05. The BP response to each drug was calculated for each measurement method by subtracting the pretreatment average from the post-treatment average. The home BP averages consisted of at least five of seven morning and evening sets of three readings taken during the week prior to the pre- and post-treatment study visits (i.e., at least 30 and up to 42 measurements prior to each visit). Multiple-variable linear regression analyses were performed to identify participant characteristics that made additive, statistically independent contributions to the prediction of systolic and diastolic BP response to each drug. In preliminary analyses, we found that higher pretreatment BP level was associated with greater BP response, as expected [20]. Because we sought to evaluate predictors that are independent of the pretreatment BP level, we first regressed out the effects of pretreatment BP level and then modeled the effects of other known predictors of BP response [10,11] as well as other variables measured at the consent and screening visit [11,17]. Final multiple-variable models were derived using a backward stepwise elimination procedure, retaining only the predictors of both systolic and diastolic BP responses to either drug. In the initial models that included race and pretreatment plasma renin activity, age was not a statistically significant predictor of BP responses and was not retained in the final models.

To determine whether known predictors of BP response depend on the method of measuring BP response, we compared models in which regression coefficients were constrained to be identical among measurement methods or allowed to differ among methods by including interactions of each predictor with the method of BP measurement. Considering the model in which the regression coefficients were identical across methods as a null hypothesis, we attempted to detect any departures indicating dependency of the predictors on method of BP measurement that would lead us to reject the "same signal" model. This analysis used PROC GENMOD in SAS, which adjusts for the correlation among the four BP response measurements within each participant.

To estimate signal-to-noise ratios, the covariance matrix of the four measured BP responses was used to estimate the signal and noise components for each method of measuring BP response after regressing out the method-specific effects of pretreatment BP level. The correlation coefficient between BP responses measured by two methods provides a dimensionless measure of how much the two responses covary (change together); the covariance between them expresses the correlation in units of the two BP responses multiplied together (mm^2^Hg) and is the variance shared between them, i.e., the signal variance. Since each pair-wise covariance provides an unbiased estimate of the signal variance, we used the average of the six pairwise covariances as the BP response signal. Subtracting the signal from the method-specific total variance provided an estimate of the method-specific error variance or noise.

We examined implications of the signal-to-noise analyses for accomplishing the goal of the PEAR and other pharmacogenomic studies. Specifically, we compared power and samples sizes required to identify a genetic polymorphism that predicts BP response when measured by each method separately and by weighted averages of the responses measured by multiple methods. The rationale for the weighted averages was to increase the signal-to-noise ratio (and power) by minimizing the error variance. Two different combinations of the measured BP responses were considered: a weighted average of all four methods and a weighted average of the office and home BP responses. The weighted average combinations were determined based on the row sums of the inverse of the inter-method covariance matrices, which provide weights that minimize the variance [21].

For the power and samples size calculations, we assumed that a genetic polymorphism with a minor allele frequency of 0.2 influences the BP response signal with an effect size that can be detected with 80% power in a sample of N = 300 at a genome-wide significance level of 5 × 10^-8^. This *P*-value was originally suggested for genome-wide association analysis of 1 million single nucleotide polymorphisms using a Bonferroni correction for multiple testing [22]. Based on the estimated signal variances, we calculated the allele effect sizes (in mmHg/allele) and the percentage of variation in the BP response (*R*^2^×100%) explained by the polymorphism. We then calculated the power to detect the polymorphism in a sample of N = 300 when the BP response is measured by each method separately and weighted averages of multiple methods, and the corresponding sample sizes required to maintain 80% power.

## Results

### Sample description

Five hundred and ninety-five study participants had complete measurements of office, home, and ambulatory daytime and nighttime BP responses (Table 1). Of these, 293 participants were randomized to atenolol (49%) and 302 to hydrochlorothiazide (51%) treatment. Mean values and relative frequencies of participant characteristics measured prior to randomization did not differ significantly between the atenolol and hydrochlorothiazide-treated groups [11] (not shown).

**Table 1:** Descriptive characteristics of study participants

	Mean ± standard deviation or N (%)
N (%)	595 (100)
Randomized to hydrochlorothiazide, N (%)	302 (51)
Age, years	49.3 ± 9.1
Male, N (%)	280 (47)
Black, N (%)	245 (41)
BMI, kg·m^-2^	30.6 ± 5.6
Hypertension duration, years	7.1 ± 7.2
Antihypertensive medication, N (%)	492 (91)
Current smoker, N (%)	69 (13)
Glucose, mg·dL^-1^	94.8 ± 10.5
Creatinine, mg·dL^-1^	0.9 ± 0.2
Serum ALT, U·L^-1^	29.1 ± 15.9
Plasma renin activity, ng·mL^-1^·hr^-1^	1.0 ± 1.2
Resting heart rate, beat·min^-1^	71.0 ± 10.2
Screening office systolic BP, mmHg	137.9 ± 13.8
Screening office diastolic BP, mmHg	89.5 ± 8.8
Pretreatment systolic blood pressure, mmHg	151.5 ± 13.8
Pretreatment diastolic blood pressure, mmHg	98.2 ± 6.3

BMI, body mass index; ALT, alanine aminotransferase; BP, mmHg. Characteristics were measured at the screening visit except pretreatment systolic and diastolic BP and plasma renin activity were measured at the end of the drug-free washout period prior to initiating atenolol or hydrochlorothiazide therapy.

### Office, home, and ambulatory BP response

Means and standard deviations of the systolic and diastolic BP responses differed among measurement methods (Table 2). For systolic BP response, office measurements had the greatest mean declines and home measurements the smallest mean declines in response to each drug (Table 2). For diastolic BP response, office measurements also had the greatest mean declines in response to each drug; ambulatory nighttime measurements had the smallest mean decline in response to atenolol and home measurements the smallest mean decline in response to hydrochlorothiazide. Correlation coefficients between the office, home, ambulatory daytime and nighttime BP responses were modest in magnitude (not shown), ranging from 0.36 to 0.71 after adjustment for differences in pretreatment BP levels (all *P *< 0.0001).

**Table 2:** Blood Pressure Responses to Monotherapy by Measurement Method

	All N = 595	Atenolol N = 293	Hydrochlorothiazide N = 302
Systolic BP Response, mmHg			
Office	-13.4 ± 14.7	-13.5 ± 15.6	-13.2 ± 13.7
Home	-8.8 ± 9.8	-8.3 ± 10.4	-9.4 ± 9.1
Ambulatory daytime	-11.5 ± 10.5	-12.2 ± 11.1	-10.8 ± 9.8
Ambulatory nighttime	-9.7 ± 12.5	-8.9 ± 12.9	-10.6 ± 12.1
Contrast *P *value	< 0.001	< 0.001	< 0.001
Diastolic BP Response, mmHg			
Office	-8.6 ± 8.9	-10.5 ± 9.4	-6.8 ± 7.9
Home	-6.6 ± 6.5	-7.8 ± 6.8	-5.3 ± 6.0
Ambulatory daytime	-7.6 ± 7.6	-9.2 ± 7.9	-6.1 ± 7.0
Ambulatory nighttime	-6.8 ± 9.5	-7.0 ± 10.0	-6.5 ± 9.0
Contrast *P *value	< 0.001	< 0.001	0.10

BP, blood pressure.

### The BP response signal and its predictors

We assessed whether the office, home, and ambulatory daytime and nighttime BP responses measure the same BP response signal by determining whether the predictors of BP response depend on the method of BP measurement (see Methods). After adjustment for pretreatment BP level, none of the predictors of BP response depended upon of the method of BP measurement (analyses not shown). For all four methods of measuring BP response, the identified predictors included race, plasma renin activity, and sex (Table 3). As expected, black race was associated with lesser systolic and diastolic BP responses to atenolol and greater responses to hydrochlorothiazide; and greater log renin was associated with greater systolic and diastolic BP responses to atenolol and lesser responses to hydrochlorothiazide [11]. Male sex was independently associated with lesser systolic and diastolic BP responses to each drug. Greater log hypertension years and greater serum ALT were each independently associated with greater systolic and diastolic BP responses to atenolol but not to hydrochlorothiazide.

**Table 3:** Multi-variable linear regression modeling of predictors of blood pressure response signal after adjustment for pretreatment blood pressure level

	BP Response to Atenolol (N = 293)		BP Response to Hydrochlorothiazide (N = 302)	
	
	Systolic β ± SE	Diastolic β ± SE	Systolic β ± SE	Diastolic β ± SE
Intercept	-14.1 ± 0.9§	-11.8 ± 0.6§	-11.8 ± 0.7§	-6.7 ± 0.5§
Race: Black	6.3 ± 1.1§	4.8 ± 0.8§	-2.9 ± 0.9‡	-2.0 ± 0.6‡
Log Plasma Renin Activity	-4.4 ± 0.5§	-2.5 ± 0.3§	0.7 ± 0.5	0.7 ± 0.3*
Sex: Male	2.3 ± 1.0*	2.9 ± 0.7§	4.2 ± 0.8§	2.7 ± 0.5§
Log Hypertension Years	1.3 ± 0.5*	1.0 ± 0.4†	-	-
Serum ALT	0.08 ± 0.03†	0.04 ± 0.02*	-	-
Model *R*^2^×100%	22%	23%	11%	16%

BP, blood pressure; β, regression coefficient; SE, standard error. Model parameters are estimated at the mean values for each quantitative predictor variable in a combined dataset used to model the predictors of office, home, ambulatory daytime and nighttime BP responses after adjustment for pretreatment BP levels. *P*-values for tests of model parameters = 0: *, ≤0.05; †, ≤0.01; ‡, ≤ 0.001; §, ≤0.0001. *R*^2^×100% is the percentage of variation in the office BP response explained by the model predictors.

### Signal-to noise-ratios

**Figure 1:**
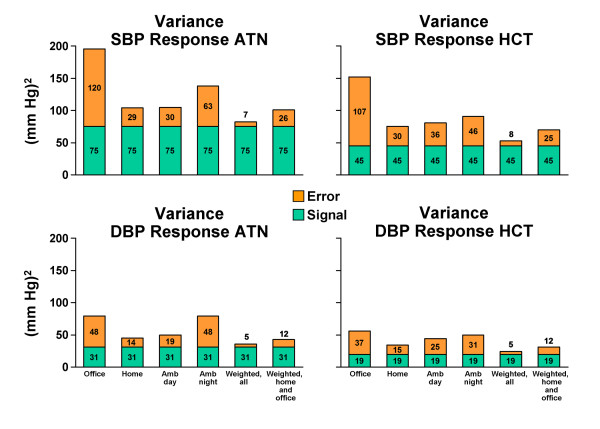
**Signal, noise, and total variances of the measured blood pressure responses to single-drug therapy with atenolol or hydrochlorothiazide and their weighted averages**.

**Figure 2:**
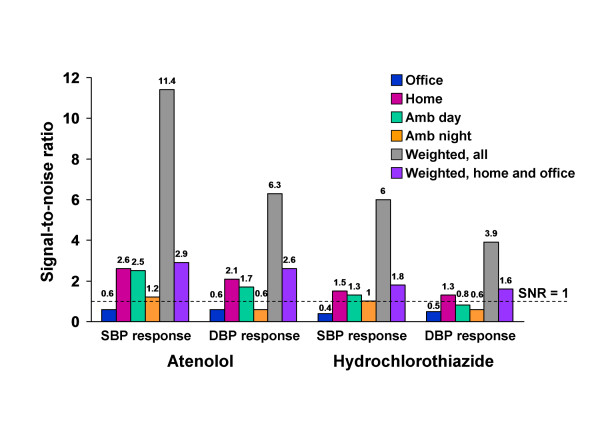
**Signal-to-noise ratios of the measured blood pressure responses to single-drug therapy with atenolol or hydrochlorothiazide and their weighted averages**.

Inferring that all four methods measure the same BP response signal, we estimated the signal variance (see Methods) and calculated the method-specific error variance (noise) and signal-to-noise ratio for each measured BP response (Figures 1 and 2). The home and ambulatory daytime BP responses had the largest signal-to-noise ratios and the ambulatory nighttime and office BP responses the smallest signal-to-noise ratios (Figure 2). The signal-to-noise ratios of the home and ambulatory daytime BP responses were similar in magnitude and up to 4-fold greater than the signal-to-noise ratios of the office and ambulatory nighttime BP responses, which were mostly less than 1 (more noise than signal). Weighted averages of all four measured BP responses improved the signal-to-noise ratios up to 4-fold compared to the home BP responses and up to 19-fold compared to the office BP responses. Weighted averages of the home and office BP responses improved the signal-to-noise ratios modestly compared to the home BP responses (by 24% at most). The weightings, provided in Additional file 1: Table S1, minimized the error variance (noise) of the average BP responses (see Methods), thereby accounting for the improvement in signal-to-noise ratios.

### Power and sample size needed to identify a genetic predictor of blood pressure response

We assumed that a genetic polymorphism with minor allele frequency of 0.2 influences the BP response signal and is detected with 80% power at genome-wide significance level of 5 × 10^-8 ^(see Methods) [22]. The T-statistic for association of the polymorphism with BP response is 6.29; 11.6% of the signal variation is explained (*R*^2^×100%); and the corresponding effect sizes (β-coefficients) in mm Hg per allele are 5.22/3.36 for the systolic/diastolic BP responses to atenolol and 4.04/2.63 for the systolic/diastolic BP responses to hydrochlorothiazide. Based on the signal and noise analyses, power to detect this polymorphism in a sample size of N = 300 declined for all methods of measuring BP response when compared to a perfect method capable of measuring only signal and no noise (Table 4). Power declined most markedly for the office BP responses (to < 5% power) and was only maintained at > 50% for the weighted averages of all four methods. Alternatively, to maintain 80% power the sample sizes would need to be increased for all methods of measuring BP response when compared to the N = 300 sample size for the perfect measurement of signal without noise (Table 4). The sample sizes increased most markedly for the office BP responses (by > 200%), but only modestly for the weighted averages of all four methods (by ≤ 26%).

**Table 4:** Power and sample sizes to detect single nucleotide polymorphism influencing BP response measured by office, home, ambulatory daytime and nighttime blood pressure

	Drug			
	
	Atenolol		Hydrochlorothiazide	
	
	Power, N = 300	N, 80% power	Power, N = 300	N, 80% power
Systolic BP response signal	80%	300	80%	300
Measurement methods				
Office	4%	780	1.5%	1013
Home	42%	416	24%	500
Ambulatory day	41%	420	18%	540
Ambulatory night	17%	552	12%	607
Weighted averages				
All methods	70%	328	61%	353
Home and office	45%	404	30%	467
Diastolic BP response signal	80%	300	80%	300
Measurement methods				
Office	5%	764	3%	884
Home	39%	426	19%	537
Ambulatory day	39%	426	19%	537
Ambulatory night	27%	484	7%	695
Weighted averages				
All methods	63%	348		379
Home and office	42%	416	26%	489

BP, blood pressure. The power and sample size estimates are for a single nucleotide polymorphism with minor allele frequency of 0.2 that influences the BP response signal with an effect size detected with 80% power in a sample of N = 300 at a genome-wide significance level of 5 × 10^-8^(see Methods). The estimates assume that the BP response signal can be measured without error.

## Discussion

Our first objective was to assess whether office, home, and ambulatory daytime and nighttime measurements of BP response to single-drug therapy measure the same BP response signal. Since different BP response signals may have different predictors, this assessment was based on determining whether known predictors of BP response [11] depend on the method of BP measurement. After adjustment for the method-specific effects of pretreatment BP level, the identified predictors of BP response were independent of the method of BP measurement. This finding supports the inference that office, home, and ambulatory daytime and nighttime BP responses measure the same BP response signal.

Our second objective was to estimate signal-to-noise ratios and compare the power to identify a genetic polymorphism predicting BP response when measured by each method separately and by weighted averages of multiple methods. Estimation of the BP response signal allowed us to also compare each method with a theoretically perfect measurement consisting of pure signal and no noise. We reasoned that greater signal-to-noise ratios would translate into greater power and smaller sample sizes required to identify a polymorphism influencing BP response to antihypertensive drug therapy. Not surprisingly, signal-to-noise ratios were greater for the home and ambulatory daytime methods, which are based on more measurements per subject and have smaller error variances, than for the office and ambulatory nighttime methods, which are based on fewer measurements per subject and have larger error variances. Particularly unsettling were the signal-to-noise ratios less than one for office and ambulatory nighttime BP responses, indicating more noise than signal for these methods. Such measurement imprecision could account for limited success in previous studies to identify predictors of office BP response [12,23] and the requirement for sample sizes in the tens of thousands for genome-wide association analyses of BP level [24]. Moreover, the profound lack of power to identify a genetic predictor of BP response in sample sizes ≤300, and the large increases in sample size required to maintain 80% power, emphasizes the need for more precise methods of measuring BP response than office BP measurements provide [25].

Although the home and ambulatory daytime BP responses provided greater power than the office and ambulatory nighttime BP responses, the estimated sample size required to maintain 80% power with either method was still in excess of the number of participants randomized to each single-drug therapy in the PEAR study (i.e., N = 400). Consequently, we pursued additional strategies to increase power by combining all of the measurements from multiple methods in a weighted average, with the weights chosen to minimize the error variance (noise) and maximize the signal-to-noise ratio of the resulting average. We provided two examples: a weighted average of measurements from all four methods and a weighted average of the home and office measurements. The latter uses the two most feasible and widely available methods of measuring antihypertensive drug responses. While both weighted averages demonstrated improvements in the signal-to-noise ratios relative to the separate methods of measuring BP response, only with the weighted average of all four methods was power maintained at 80% without an increase in sample size exceeding the N = 400 randomized to each single-drug therapy in the PEAR study. These signal and noise analyses, power calculations, and sample size estimates based on the PEAR study emphasize the "make-or-break" contribution that precision in measurement of the phenotype can make to success of genome-wide association studies [26].

Given our interest in the PEAR study to identify new predictors of BP response, several additional results of our analyses are noteworthy. First, although signal variances were greater for the systolic than the diastolic BP responses, the error variances were also greater and the signal-to-noise ratios differed little between the systolic and diastolic BP responses. This finding suggests that neither phenotype affords greater opportunity than the other to identify its predictors. This suggestion is supported by the finding that each known predictor was a statistically significant predictor of both systolic *and *diastolic BP responses (Table 3). Second, greater signal and signal-to-noise ratios for the BP responses to atenolol might suggest greater predictability of BP response to atenolol than to hydrochlorothiazide. This suggestion is supported by the finding that two identified predictors of BP response to atenolol were not predictors of BP response to hydrochlorothiazide (Table 3). Third, male sex was associated with lesser systolic and diastolic BP responses to both atenolol and hydrochlorothiazide in this study and to hydrochlorothiazide in a previous pharmacogenetic study [12]. Male sex was also previously associated with lesser responses to quinapril [27] and candesartan [13]. To our knowledge, this apparently consistent association of male sex with lesser BP response to drugs from different pharmacological classes has not been previously recognized.

Despite many studies of antihypertensive drugs conducted since the 1950s, few patient characteristics have been identified that predict inter-individual differences in BP responses. Methods that reduce the error in measuring blood pressure response, especially weighted averages of the responses measured by multiple methods, improve signal-to-noise ratios and provide greater power to identify the predictors of response in smaller sample sizes. Their incorporation in the design of pharmacogenomic studies such as the PEAR study will be critical to success in identifying novel genetic polymorphisms that improve the ability to predict BP response to antihypertensive drug therapy.

## Conclusion

Since different methods of measuring BP response to antihypertensive drug therapy measure the same signal, weighted averages of the BP responses measured by multiple methods minimize measurement error and optimize power to identify genetic predictors of BP response.

## Competing interests

The authors declare that they have no competing interests.

## Authors' contributions

ST and GS participated in the design of the study, collected and analyzed the data, and drafted and revised the manuscript. AB, JG, RCD participated in the design of the study, collected the data and participated in revision of the manuscript. JJ conceived and designed the study, collected the data, and participated in revision of the manuscript. EB participated in design of the study and revision of the manuscript. KB participated in design of the study, analyzed the data, and participated in drafting and revision of the manuscript. All authors read and approved the final manuscript

## Supplementary Material

Additional file 1 **Additional file 1: Table S1**. Esimated weights for calculation of minimum variance weighted average blood pressure responses. Click here for file
