# Supplementary material for: Power to identify a genetic predictor of antihypertensive drug response using different methods to measure blood pressure response
Source: J Transl Med. 2012 Mar 13;10:47. doi: 10.1186/1479-5876-10-47 (PMC3342146; doi:10.1186/1479-5876-10-47)
Supplement: Additional file 1 — Table S1. Esimated weights for calculation of minimum variance weighted average blood pressure responses. [file 1479-5876-10-47-S1.DOC]

Supplementary Table S1. Esimated weights for calculation of minimum variance weighted average blood pressure responses

|  | BP Response to Atenolol | | BP Response to Hydrochlorothiazide | |
| --- | --- | --- | --- | --- |
|  | Systolic | Diastolic | Systolic | Diastolic |
| Average of all four methods |  |  |  |  |
| Home | 0.45 | 0.48 | 0.45 | 0.48 |
| Ambulatory daytime | 0.40 | 0.35 | 0.31 | 0.27 |
| Ambulatory nighttime | 0.15 | 0.10 | 0.18 | 0.16 |
| Office | 0.00 | 0.07 | 0.05 | 0.09 |
| Average of three methods |  |  |  |  |
| Home | 0.50 | 0.51 | 0.49 | 0.52 |
| Ambulatory daytime | 0.49 | 0.42 | 0.44 | 0.36 |
| Office | 0.01 | 0.07 | 0.07 | 0.12 |
| Average of two methods |  |  |  |  |
| Home | 0.84 | 0.79 | 0.81 | 0.74 |
| Office | 0.16 | 0.21 | 0.19 | 0.26 |

The weighted average combinations were determined based on the row sums of the inverse of the inter-method covariance matrices, which provide weights that minimize the variance (see Methods).
